# Whole-transcriptome analysis of periodontal tissue and construction of immune-related competitive endogenous RNA network

**DOI:** 10.1186/s12903-022-02401-0

**Published:** 2022-08-31

**Authors:** Quanquan Zhao, Jing Wen, Xiangying Ouyang, Jianru Liu, Wenyi Liu, Shengnan Zhang, Peiying Lv, Xinzhe Lou

**Affiliations:** Department of Periodontology, National Clinical Research Center for Oral Diseases, National Engineering Laboratory for Digital and Material Technology of Stomatology, Beijing Key Laboratory of Digital Stomatology, School and Hospital of Stomatology, Peking University, 22 Zhongguancun South Avenue, Haidian District, Beijing, 100081 People’s Republic of China

**Keywords:** Periodontitis, Immunomodulation, Noncoding RNA, Gene expression profiling, RNA-seq

## Abstract

**Background:**

In periodontitis, noncoding RNAs may play a regulatory role in the immune microenvironment through competitive endogenous RNA. We aimed to profile noncoding RNA expression and construct immune-related ceRNA network in periodontitis.

**Methods:**

Five inflamed periodontal tissue and five healthy gingivae were collected for whole-transcriptome sequencing. Differential gene, functional enrichment, and protein–protein interaction network analysis were performed to explore the function of differentially expressed genes. CIBERSORTx was used to analyze level of immune cell infiltration in the periodontal tissue. An immune-related competitive endogenous RNA network was constructed and expression of key regulators in the network was validated.

**Results:**

Compared with healthy gingiva, 200 mRNAs, 90 long noncoding RNAs, 65 microRNAs, and 518 circular RNAs were differentially expressed, and cell chemotaxis was significantly enhanced in inflamed periodontal tissue. Immune cell infiltration analysis showed that neutrophils, macrophages M1, T follicular helper cells, and naive B cells were significantly increased in periodontitis. Key regulators including JUN, FOS, THBS1, KLF2, WIF1, were identified and their expression was then validated.

**Conclusion:**

We constructed an immune-related competitive endogenous RNA network in periodontal tissue, which provided new insights into immune homeostasis in periodontitis and laid a foundation for further study of noncoding RNAs. Key regulators in this network may be promising targets for future periodontitis treatment.

**Supplementary Information:**

The online version contains supplementary material available at 10.1186/s12903-022-02401-0.

## Introduction

Periodontitis is a chronic infectious inflammatory disease driven by reciprocally reinforced interactions between the dysbiotic microbiome and dysregulated immunity [1, 2]. Many studies have found that expression of specific genes play a role in the pathogenesis of periodontitis and its susceptibility [3–5]. Therefore, comprehensive omics researches could provide us more information about the formation and progress of the diseases.

Noncoding RNAs (ncRNAs), including long noncoding RNAs (lncRNAs), microRNAs (miRNAs), and circular RNAs (circRNAs), participate in multiple biological processes and in the pathogenesis of diseases, such as tumors, Alzheimer's disease and other inflammatory diseases [6]. The competitive endogenous RNA (ceRNA) hypothesis describes an intricate interplay among diverse RNA species. miRNAs complementarily pair with mRNAs through miRNA response elements (MREs), decreasing mRNA expression levels. lncRNAs or circRNAs also have MREs, to regulate mRNA expression levels through competitive binding with miRNA [7]. Several studies have constructed lncRNA-related ceRNA networks in periodontitis [8]. It has been reported that lncRNA MIAT and the MIAT-based ceRNA network may regulate the immune response during the progression of periodontitis [9]. Besides, lncRNA FGD5-AS1 were reported to be involved in the pathogenesis of periodontitis through ceRNA [10].

The complex coordination of inflammation and the immune response in the periodontal tissue is important in periodontitis. However, there are only a few studies on ceRNA related to immune regulation in periodontitis, and most studies only focus on several specific lncRNAs. Comprehensive analysis of immune-related ncRNAs and ceRNA network including lncRNAs and circRNAs has not been reported. Therefore, the objective of our study was to comprehensively profile the differential expression of mRNAs, lncRNAs, circRNAs and miRNAs in the inflamed periodontal tissue (IPT) and healthy gingiva through whole-transcriptome sequencing, and construct an immune-related ceRNA network in periodontitis, to identify key regulators which may serve as potential therapeutic targets.

## Material and methods

All methods were performed in accordance with the relevant guidelines and regulations.

### Sample collection

Tissue samples were collected during periodontal surgery at the Peking University Hospital of Stomatology from May 2020 to April 2021. All the participants gave written informed consent.

The inclusion criteria were as follows:

- Patients aged 18–65 years, systemically healthy, who agreed to participate in the trial and had finished non-surgical therapy.
- The IPT was collected from the operative site with pocket depth (PD) ≥ 6 mm and bleed index (BI) > 2 in patients diagnosed stage III and grade C periodontitis.
- Healthy gingival tissue was collected from patients who underwent crown lengthening, PD ≤ 3 mm and BI ≤ 2, and showed no alveolar bone loss on radiography.

The exclusion criteria were as follows:

- Patients with acute periodontal disease.
- Patients received antibiotics or underwent periodontal surgery in the past 3 months.
- Smokers, and pregnant or lactating women.

Clinical measurements of PD, BI, clinical attachment loss (CAL), gingival recession (REC) were recorded. The tissue samples collected were rinsed with 0.9% normal saline, immediately frozen in liquid nitrogen, and stored at − 80 °C. In total, 20 periodontitis and 20 healthy samples were collected, five from each group were used for sequencing and 15 were used for validation.

### High-throughput sequencing and data processing

Total RNA was extracted from five IPT and five healthy gingivae, and sequenced using the BGISEQ-500 platform (Huada Gene Technology). Sequencing data were filtered using SOAP nuke, and clean reads of lncRNAs and mRNAs were mapped to reference the genome using HISAT2. Stringtie was used to assemble and quantify transcripts. Small RNA clean reads were aligned to reference the genome (Hg19) using Bowtie 2 and quantified with FeatureCounts. Since single prediction software programs often have certain limitations, we used two programs, i.e., CIRCexplorer2 and find_circ, to identify circRNAs, and their intersection was retained for further analysis. All expression profiles were used for principal component analysis.

### Differential gene analysis and functional enrichment analysis

DESeq2 was used for differential expression analysis, mRNAs, genes with *P* ≤ 0.05, FDR ≤ 0.05, and |log2FC (fold change)|≥ 1 were identified as differentially expressed genes (DEGs). GO and KEGG enrichment analyses were performed with clusterProfiler. Then, we used the MSigDB C5 GO and MSigDB C2 KEGG gene sets for gene set enrichment analysis (GSEA) [11]. miR-Path was used to perform enrichment analysis of differentially expressed miRNAs.

### Construction of the protein–protein interaction network

We used the STRING database to identify the protein–protein interaction (PPI) between differentially expressed mRNAs, with confidence > 0.7. The PPI network was then imported into Cytoscape for topology property analysis. Cytohubba were used to identify hub genes.

### Immune cell infiltrations analysis

All expression profiles were imported to CIBERSORTx for immune cell infiltrations analysis. CIBERSORTx is widely used in immune cell infiltration analysis for bulk RNA-sequence data. The correlation coefficient between differentially expressed lncRNA/circRNA and the expression of characteristic genes in immune-infiltrated cells was calculated, and ncRNA/circRNA with correlation > 0.8 were selected as immune-related.

### ceRNA network construction

miRNA–mRNA interactions were predicted using miRWalk 2.0, which uses 12 predicted algorithms, and target genes predicated by six algorithms were maintained for further analysis. The lncRNA‐miRNA interactions were extracted from miRcode and starBase, and the circRNA-miRNA interactions were retrieved from the circBank database. Expression of lncRNAs, mRNAs, and circRNAs were used for co-expression analysis. Pearson’s correlation coefficients ≥ 0.95 were considered to indicate co-expression. The ceRNA network was constructed by integrating all validated interactions and co-expression pairs. On this basis, we constructed an immune-related ceRNA network combined with the immune-related lncRNAs/circRNAs, and immune-related mRNAs from ImmPort database.

### Validation of expression patterns

Total RNA from 15 inflamed periodontal tissue and 15 healthy gingivae was isolated using TRIzol® Reagent. The ABScript II cDNA Fist-Strand Synthesis Kit (ABclonal Technology Co., Ltd, Wuhan, China) was used for reverse transcription to synthesize cDNA. RT–qPCR was conducted using qPCR SYBR® Green Master Mix (Roche Holding AG, Basel, Switzerland) on an ABI Q3 system (Applied Biosystems, Foster City, CA, USA). GAPDH was used as endogenous reference. All primer sequences were synthesized by Sangon Biotech. mRNA expression was determined using the ΔΔCT method. Difference between two groups were evaluated with t tests. Statistical analyses were performed with SPSS 22.0 (IBM, Armonk, NY, USA).

## Results

### Clinical characteristics and transcriptome profile

Demographic and clinical parameters of patients included in the study are listed in Table 1. The mean PD, BI, and CAL of the periodontitis group were significantly higher than those in the healthy group (*p* < 0.01). Based on the transcriptome, we identified 16,880 lncRNAs, 19,962 mRNAs and 2654 miRNAs. For circRNAs, CIRCexplorer2 predicted 31,608 circRNAs and find_circ predicted 27,856, the intersection of the two was used for subsequent analyses (Additional file 1: Fig. s1).

**Table 1:** Demographic and clinical parameters of patients

	Periodontitis(n = 5)	Healthy gingiva(n = 5)	*p*-value
Age	34.7 ± 5.21	31.2 ± 5.89	0.244
Gender			
Male	2	3	*p* < 0.01
Female	3	2	
Pocket depth(PD; mm)	6.67 ± 0.33	2.80 ± 0.20	*p* < 0.01
Bleeding index(BI)	1.33 ± 0.33	0.60 ± 0.24	*p* < 0.01
Clinical attachment loss(CAL; mm)	7.50 ± 0.29	0.60 ± 0.40	*p* < 0.01
Recession(REC; mm)	0.83 ± 0.60	0.41 ± 0.80	0.241

Principal component analysis showed that the two groups could be divided well in 95% confidence intervals, suggesting that the transcriptome in IPT differs from healthy gingiva (Additional file 1: Fig. s1).

### Differential expression gene analysis of RNA-seq data

In total, 90 lncRNAs, 200 mRNAs, 65 miRNAs and 518 circRNAs were found to be differentially expressed. Among them, 146 mRNAs were upregulated and 54 downregulated; 44 lncRNAs were upregulated and 46 downregulated; 41 miRNAs were upregulated and 24 downregulated; and 163 circRNAs were upregulated and 355 downregulated (Additional file 1: Fig. s1).

### Functional enrichment analysis

**Fig. 1:**
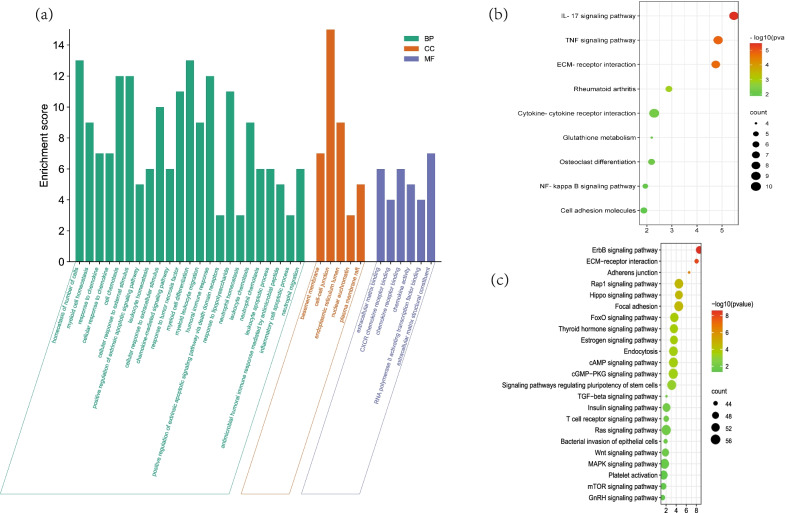
Functional enrichment analysis. **a** Top significantly enriched GO terms, BP: biological process, CC: cellular component, MF: Molecular Function, **b** KEGG pathway analysis. Top 9 significantly enriched pathways in KEGG enrichment analysis. **c** miRNA KEGG pathway analysis

GO analysis showed that 117 biological processes, 6 molecular functions, and 5 cellular components were significant enriched (*P* < 0.05). The top significantly enriched terms associated with our study are shown in Fig. 1a. The DEGs were mainly involved in cell chemotaxis. In the KEGG pathway analysis, 105 pathways were enriched, and 16 were significantly so. The results revealed that the DEGs were mainly enriched in the IL-17 signaling pathway, TNF signaling pathway, cytokine-cytokine receptor interaction, and extracellular matrix receptor interaction (Fig. 1b). miRNA enrichment analysis showed target genes of differently expressed miRNAs associated with the Hippo signaling pathway, FoxO signaling pathway, cAMP signaling pathway, TGF-β signaling pathway, and Wnt signaling pathway. In addition, the signaling pathways regulating stem cells pluripotency was significantly enriched (Fig. 1c).

The GSEA results of C5 gene sets showed that the T cell receptor complex, odontogenesis of dentin-containing teeth, P38MAPK cascade, neutrophil chemotaxis, chemokine activity, CXCR chemokine receptor binding, biomineralization, and extracellular matrix binding were significantly upregulated in the IPT. The results of the C2 gene set showed that cytokine-cytokine receptor interaction, chemokine signaling pathway, TGF-β signaling pathway, P53 signaling pathway, Wnt signaling pathway, extra cellular matrix (ECM)-receptor interaction, focal adhesion, and cell adhesion molecule were significantly upregulated (Additional file 1: Fig. s2).

### Construction of the PPI network

**Fig. 2:**
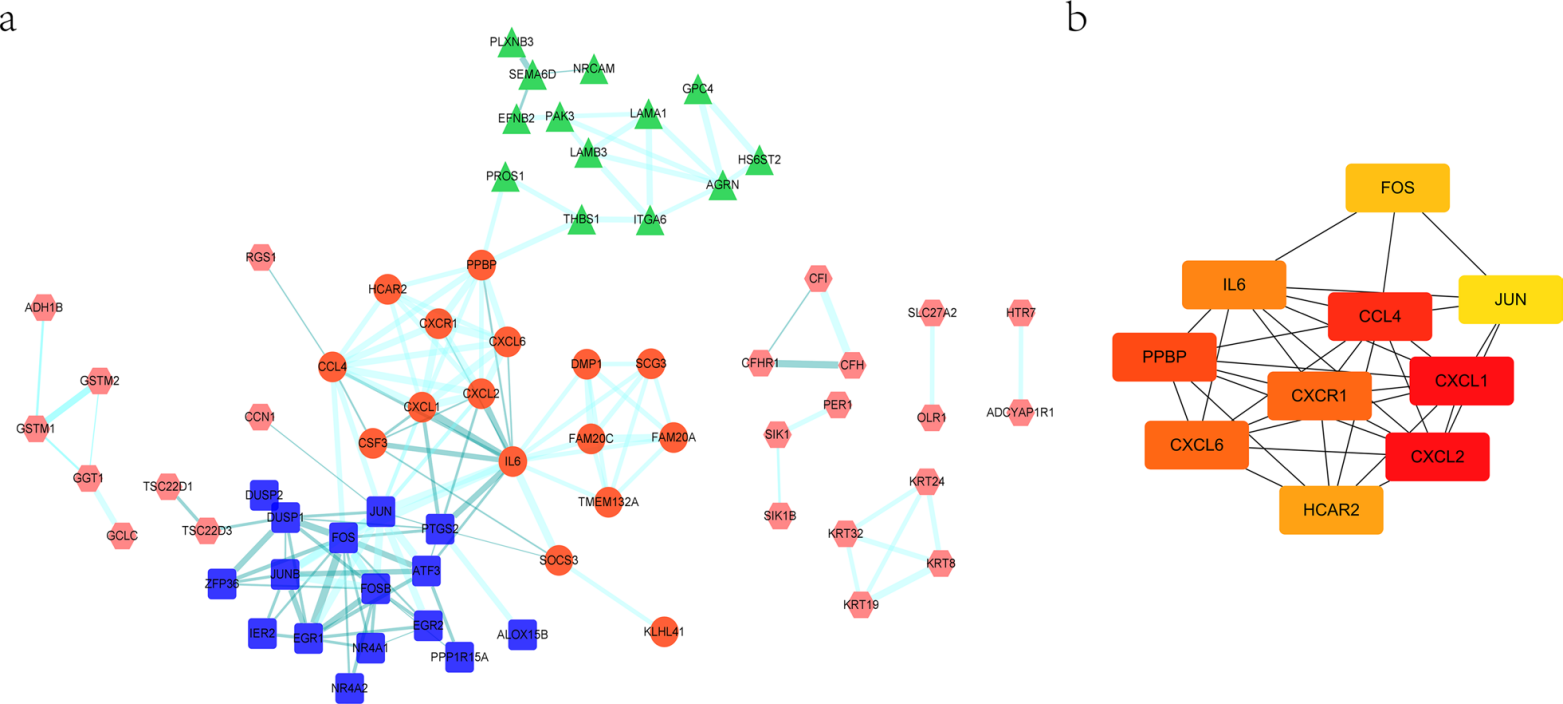
PPI network analysis. **a** PPI shows interactions between mRNAs. **b** Top 10 hub gene selected by MCC

The PPI network was constructed (Fig. 2a), and clustered into four clusters using the k-means clustering method. Cluster 1 included chemokine-and cytokine-related genes such as *CXCL6, IL6,* and *CXCR1*. Cluster 2 included transcription factors *FOS, JUN, FOSB*, and others. Cluster 3 included *LAMA1, LAMB3,* and others, located on the cell membrane and mediating extracellular matrix interactions. The top 10 DEGs with maximal clique centrality were selected as hub genes (Fig. 2b) that may play a critical role in immune homeostasis in IPT.

### Immune cell infiltrations analysis

**Fig. 3:**
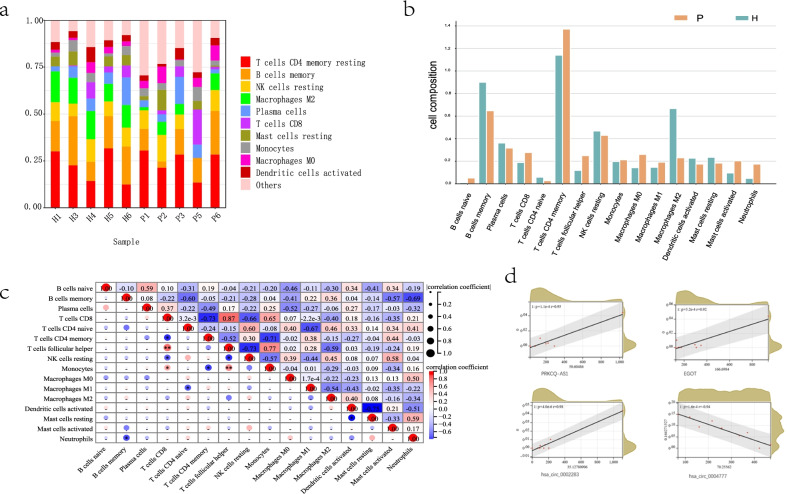
Results of immune cell infiltrations analysis. **a** Proportion of immune cells in each sample. **b** Comparison of immune cells infiltration of IPT and healthy gingiva. **c** heatmap of immune cell infiltration correlation, blue: negative correlation, red: positive correlation, *p < 0.05, **p < 0.01 **d** Scatter plot shows the correlation relationship between the 2 lncRNAs and 2 circRNAs with the highest correlation r: correlation coefficient

Immune infiltrations analysis showed 16 types of immune cell infiltrates (Fig. 3a). Lymphocytes, especially CD4 + memory T cells and B memory cells accounted for the highest proportion, followed by natural killer cells and macrophages. Compared with the healthy gingiva, the proportion of neutrophils, macrophages M1, T follicular helper cells (Tfh), and naive B cells in the IPT was significantly higher, and that of macrophages M2 was significantly lower (Fig. 3b).

The correlation analysis of 16 types of immune cells showed that monocytes, CD8 + T cells and Tfh cells were significantly positively correlated, suggesting a synergistic role. CD8 + T cells were negatively correlated with CD4 + memory T cells and nature killer cells (Fig. 3c).

The correlation analysis showed 20 lncRNAs and 146 circRNAs related to immune infiltration. lncRNA–*EGOT* and *PRKCQ-AS1*, and circRNA– *hsa_circ_0002283* and *hsa_circ_0004777* had the highest correlation (Fig. 3d).

#### Construction of ceRNA network

**Fig. 4:**
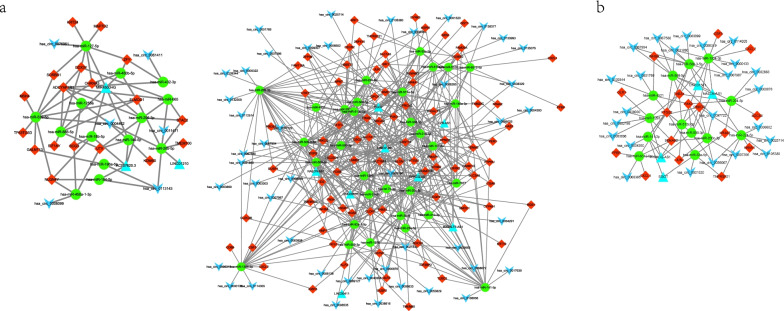
ceRNA network **a**. overall lncRNA/circRNA-miRNA-mRNA ceRNA network. **b**. immune-related lncRNA/circRNA-miRNA-mRNA ceRNA network

In total, 196 nodes (99 mRNAs, 43 miRNAs, 11 lncRNAs, and 45 circRNAs) and 502 edges were included in the ceRNA network (Fig. 4a). Based on the ceRNA network, an immune-related ceRNA network was constructed consisting of 57 nodes (4 lncRNAs, 13 miRNAs, 16 mRNAs and 24 circRNAs) and 95 edges (Fig. 4b). Five miRNAs were recognized as hub gene, including *miR-141-3p, miR-508-3p, miR-1304-3p, miR-1293,* and *miR-33a-5p* (Fig. 4c).

### Validation of expression patterns

**Fig. 5:**
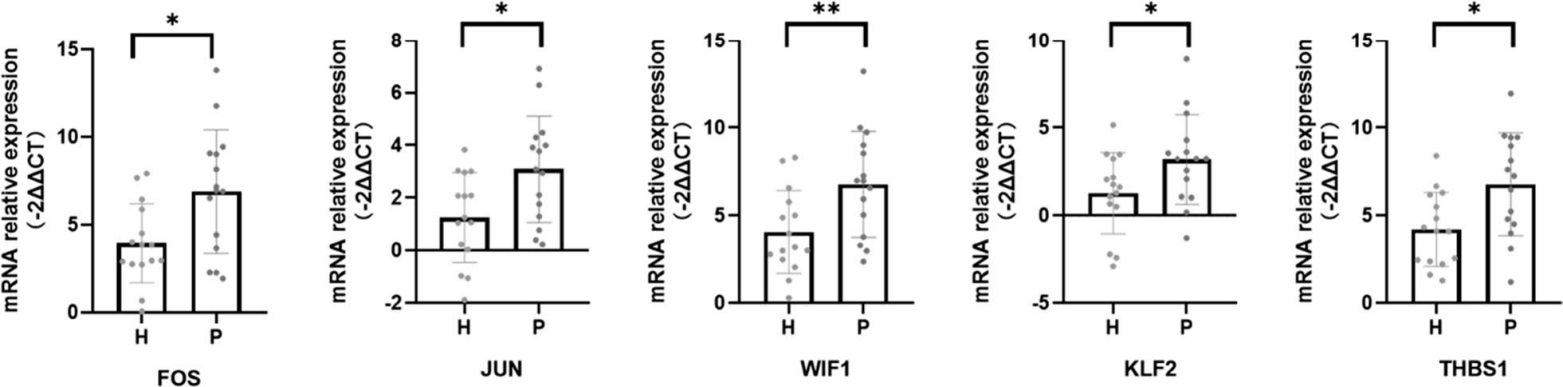
Validation of mRNA relative expression by RT–qPCR. *p < 0.05, **p < 0.01

Five mRNAs, i,e., *FOS, JUN, KLF2, WIF1,* and *THBS1*, for further validation via RT-qPCR on 15 periodontitis and 15 healthy gingivae samples. The results showed higher mRNA relative expression of *FOS, JUN, KLF2, THBS1,* and *WIF1* in the IPT (Fig. 5).

## Discussion

To our knowledge, this was the first study to perform whole-transcriptome sequencing on IPT and healthy tissue, and described the expression profile of mRNAs, lncRNAs, miRNAs and circRNAs at the same time. Zou et al. [12] and Li et al. [13] only detected lncRNAs expressions in periodontal tissues of patients with chronic periodontitis and aggressive periodontitis [12, 13]. Yu et al. [14] and Stoecklin-Wasmer et al. [15] detected the expression of circRNAs and miRNAs, and mRNAs and miRNAs repectively. Besides, all samples collected in our study were from deep intrabony defect of patients with periodontitis (Stage III Grade C), which could reflect the typical transcriptome in IPT.

Based on the whole-transcriptome analysis, we comprehensively constructed the immune-related ceRNA network in periodontitis. A previous study analyzed the transcriptome of gingiva from peri-implantitis, periodontitis, and healthy patients, and compared the difference of ceRNA network between periodontitis and peri-implantitis [16]. Lin et al. also analysis the ceRNA network in periodontitis using dataset in GEO database [8]. These studies only described the lncRNA-related ceRNA network, but circRNAs were not included.

Function enrichment analysis showed cell chemotaxis was significantly enriched. Besides, hub genes in the PPI network were mainly of chemokines and chemokine receptors. Another interesting finding of GO analysis was that tooth mineralization, and biomineralization were significantly upregulated in IPT. It has been reported that there are kinds of stem cells in IPT, which may be related to the function of these stem cells.

In our study, whole-transcriptome expression data and CIBERSORTx were used for Immune cells infiltration analysis, revealing the proportion of immune cells in periodontal tissue. It has showed that the proportion of neutrophils was not high and the highest proportion immune cell are lymphocytes. The results are consistent with the immune cell infiltration analysis by Li et al. [17]. Single-cell sequencing could directly obtain the proportion of different kinds of cells in the tissue, which is more accurate than immune cell infiltration analysis. Recently single-cell sequencing analysis of periodontal tissue also showed that neutrophils accounted for only 0.2% in healthy, and 0.8% in periodontitis samples, far lower than plasma cells (about 10%-30%) and T lymphocytes (about 20%-25%) [18]. Previous studies have confirmed that the main types of immune cells in advanced periodontitis were B cells and plasma cells, but the role of naive B cells in periodontitis has received little attention. We found the proportion of naive B cells was higher in IPT, although previous flow cytometry analysis showed few naive B cells (< 8%) in periodontal tissues [19]. However, a previous study found no significant difference in the number of naive B cells between chronic periodontitis, aggressive periodontitis and healthy group by flow cytometry [20]. In fact, the role of naive B cell in periodontal tissue is still unclear and more research is needed.

*JUN, FOS, KLF2, THBS1* and *WIF1* were identified as key regulator in periodontitis and validated to be highly expressed in IPT through RT-qPCR. We identified these 5 genes as key regulators according to function enrichment analysis and PPI network. We identified 10 hub genes in the PPI network, however, most of them are cytokines and chemokines, apart from *JUN and FOS,* which have been studied a lot. JUN and FOS are subunits of transcription factors AP-1. Activation of toll-like receptors (TLRs) stimulates the production of multiple cytokines, and eventually activates AP-1 [21]. M1 macrophages regulate TLR4/AP1 and promote alveolar bone destruction in periodontitis [22]. A deep learning-based autoencoder predicted *FOS* and *JUN* to be critical immunosuppression genes and mediate immune suppression in periodontitis [23]. *WIF1*, 29-fold up-regulated in IPT, could directly interacts with Wnt ligands and may be key to the inhibition of Wnt signaling in IPT. *KLF2* plays a key role in the activation of immune cells and participates in inflammatory diseases by regulating the NF-κB pathway. Besides, *KLF2* regulates osteoclast generation and inhibits PDSLCs osteogenic differentiation, thus participating in bone destruction [24]. *THBS1* is an important mediator involved in the chemotactic function of neutrophils and monocytes [25].

We identified 20 immune-related lncRNAs, and *EGOT* and *PRKCQ-AS1* were the top two. *EGOT* modulates the PI3K/AKT, MAPK, and NF-κB pathways to activate inflammation [26]. After LPS and TNF-α stimulation, the expression of EGOT in THP-1 and CD4, CD8 + T cells increased. *PRKCQ-AS1,* has not been studied in periodontitis, and our analysis showed that PRKCQ-AS1 may act as sponge of *miR-141, miR-6512, miR-513c*, and regulate the expression of *CXCL1, PTGS2* (COX-2), *THBS1* and *PRKCQ*.

As the central molecules of the ceRNA network, miRNAs participate in maintaining periodontal homeostasis. *miR-146a* and *miR-17* regulate the osteogenic differentiation of PDSLCs in an inflammatory environment and miR-34a, miR-146a, and miR-223 inhibit osteoclast differentiation [27]. Topological analysis of the ceRNA network showed that *miR-141-3p, miR-1304-3p, miR-1293*, and *miR-33a-5p* were hub miRNAs. Among them, the plasma derived *miR-1304-3p* exosome was down-regulated in periodontitis, and returned to normal after periodontal treatment, suggesting *miR-1304-3p* may be involved in the regulation of periodontal inflammation [28]. *miR-1293* directly bind to *IL-6* mRNA and inhibit *IL-6* expression [29]. *miR-33a-5p* can target the NF-κB pathway and Wnt/β-catenin pathway to regulate immune responses and affect cell proliferation, migration, and other biological processes [30].

circRNAs may play a key role in periodontal homeostasis. However, the related research in its infancy. Most studies on the role of circRNAs in periodontitis have mainly focused on osteogenic differentiation, or cell proliferation of PDLCs. In our study, 518 circRNAs were differentially expressed, and 146 were immune-related. Next, we will conduct further validation to clarify how these circRNAs regulate the periodontal inflammatory process.

Our study identified the above key regulators in periodontitis through bioinformatics analysis. Further experimental studies are needed to verify the exact role of the identified ceRNA network in IPT. In addition, the sample size included in our study was only 5, in the future, with the development of technology and reduction of cost, we would further expand the sample size for more accurate ncRNA expression data in IPTs.

## Conclusion

To our knowledge, this was the first study to delineate the expression profiles of ncRNAs and mRNAs in IPT, and too reveal the characteristics of immune cell infiltration in IPT. An immune-related ceRNA network was constructed. We confirmed that *JUN, FOS, KLF2, THBS1, WIF1* and *EGOT, PRKCQ-AS1* were highly expressed in IPT. Our study provided new insights into immunity homeostasis in periodontitis and laid a foundation for future research on ncRNAs.

## Supplementary Information

**Additional file 1**. **Figure s1.** (a) A Venn diagram that shows the number of circRNAs indentified by CIRCexplorer2 and find_circ; (b) Principal component analysis. Red points: healthy group samples; blue points: periodontal granulation tissue group, CI: 95% (c) volcano map shows differentially expressed genes The X axis represents log2FoldChange, the Y axis represents -log10(padj). **Figure s2.** GSEA results.

## Data Availability

The datasets generated during the current study are available in NCBI GEO database and the id was PRJNA815378.
