# Supplementary material for: Whole-transcriptome analysis of periodontal tissue and construction of immune-related competitive endogenous RNA network
Source: BMC Oral Health. 2022 Aug 31;22:370. doi: 10.1186/s12903-022-02401-0 (PMC9429583; doi:10.1186/s12903-022-02401-0)
Supplement: Supplementary file 1 — Additional file 1. Figure s1. (a) A Venn diagram that shows the number of circRNAs indentified by CIRCexplorer2 and find_circ; (b) Principal component analysis. Red points: healthy group samples; blue points: periodontal granulation tissue group, CI: 95% (c) volcano map shows differentially expressed genes The X axis represents log2FoldChange, the Y axis represents -log10(padj). Figure s2. GSEA results. [file 12903_2022_2401_MOESM1_ESM.docx]

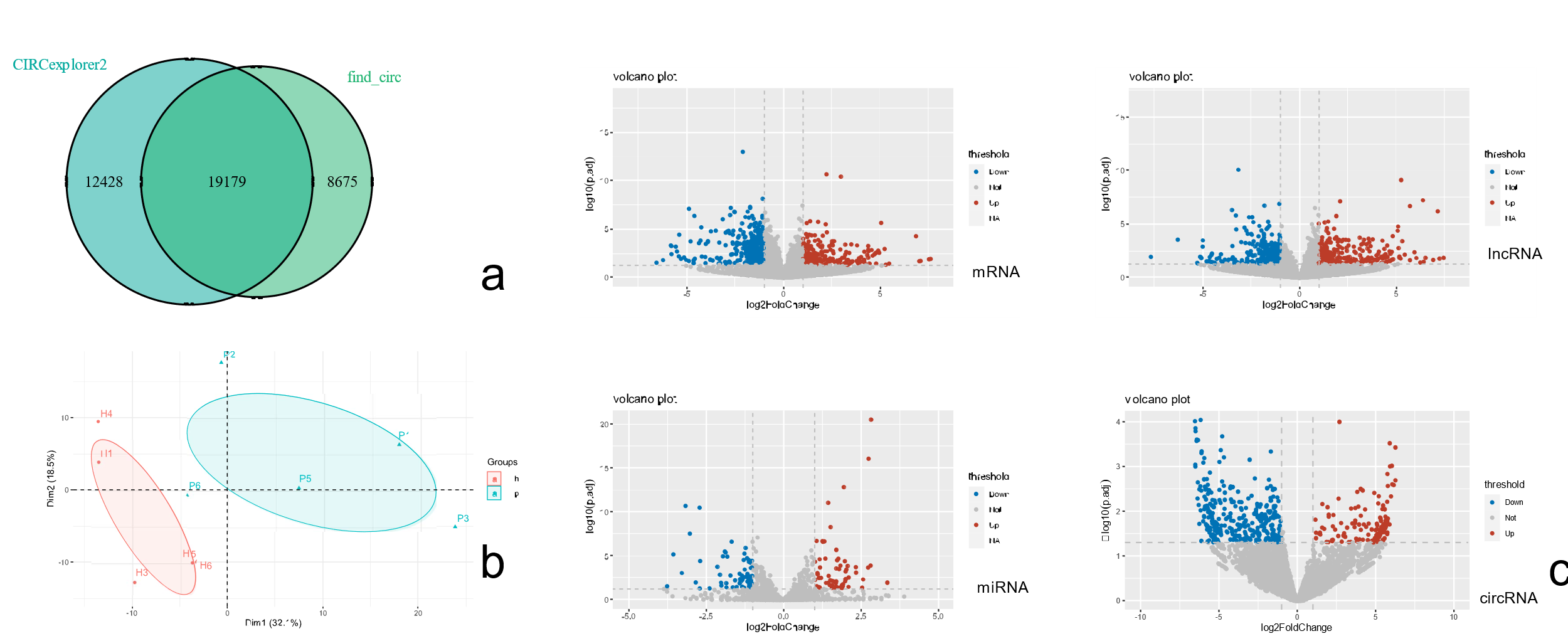


Figure s1 (a) A Venn diagram that shows the number of circRNAs indentified by CIRCexplorer2 and find_circ; (b) Principal component analysis. Red points: healthy group samples; blue points: periodontal granulation tissue group, CI:95% (c) volcano map shows differentially expressed genes The X axis represents log2FoldChange, the Y axis represents -log10(padj). Red dots: upregulated genes, blue dots :down-regulated genes, grey: non-differentially expressed genes.


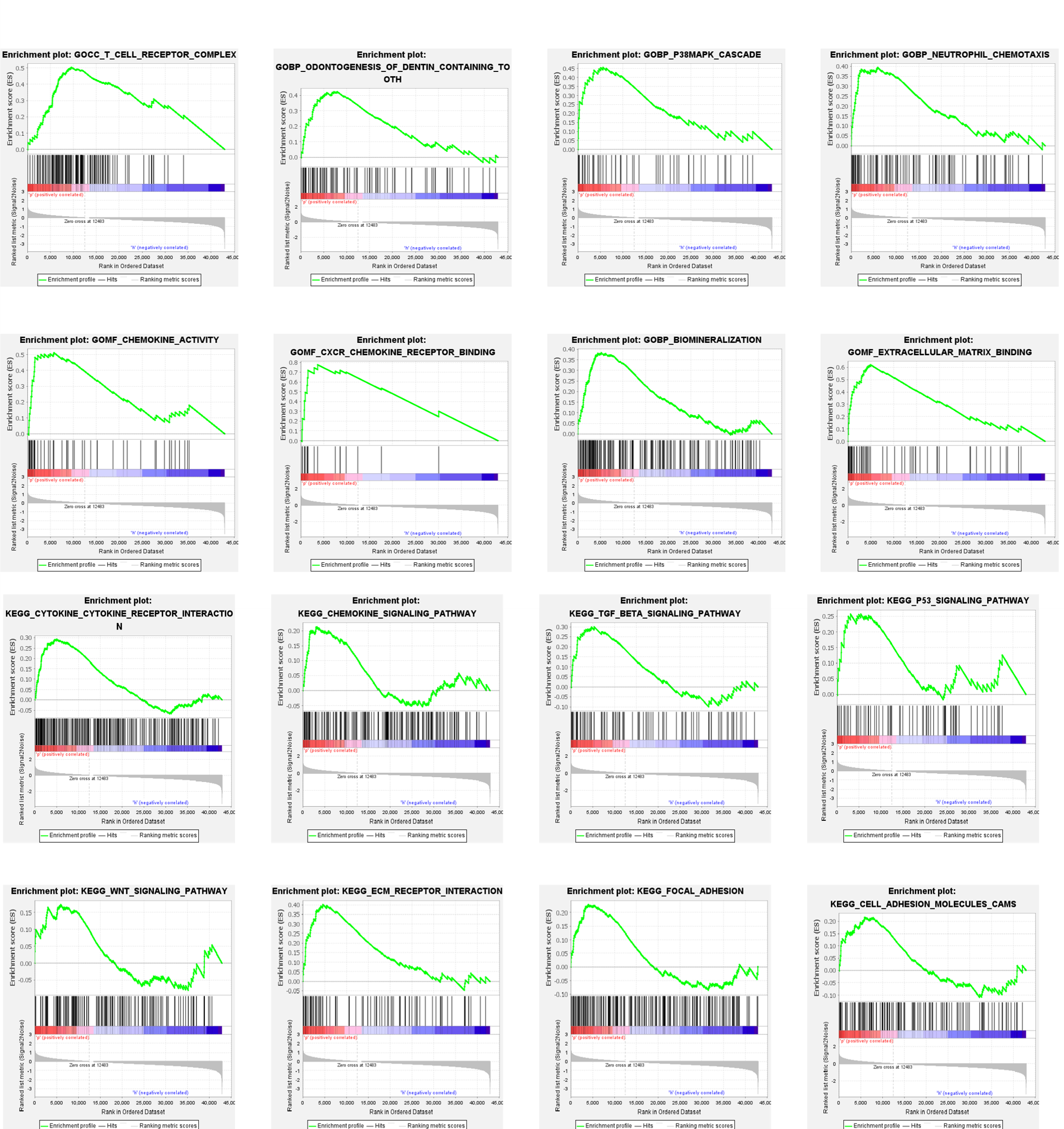


Figure s2 GSEA results
